# Correction to “Phytochemical Composition and Antioxidant and Anti‐Inflammatory Activities of Iningai Aboriginal Medicinal Plants From Central Queensland, Australia”

**DOI:** 10.1155/sci5/9869575

**Published:** 2026-07-08

G. Turpin, D. Crayn, S. Thompson, K. Yeshi, P. Wangchuk, “Phytochemical Composition and Antioxidant and Anti‐Inflammatory Activities of Iningai Aboriginal Medicinal Plants From Central Queensland, Australia,” *Scientifica*, 2026, 5727253, https://doi.org/10.1155/sci5/5727253.

In the article, the authors have identified an error in Figure 2, in which the incorrect image was selected for panel F during figure preparation. The correct Figure 2 is given below:

**FIGURE 2:**
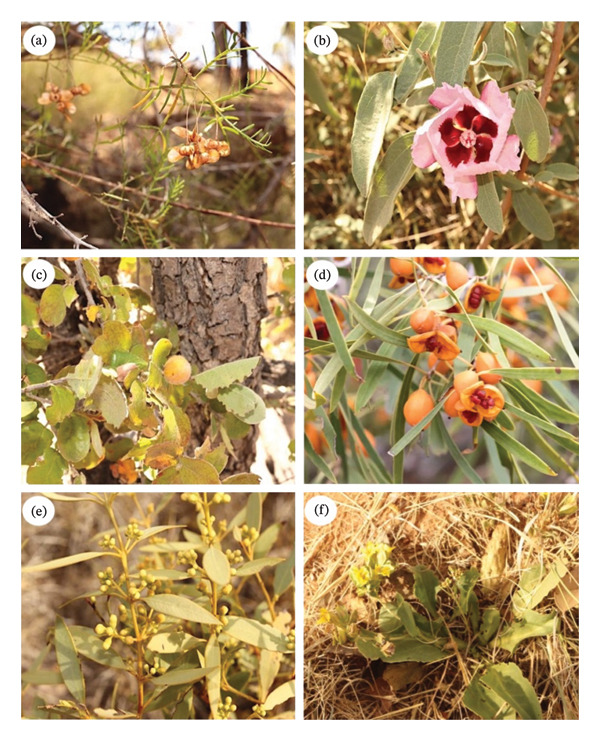
Medicinal plant species included in the study with their aerial parts: (a) *Dodonaea tenuifolia*; (b) *Gossypium australe*; (c) *Petalostigma pubescens*; (d) *Pittosporum angustifolium*; (e) *Santalum lanceolatum*; (f) *Velleia macrocalyx*. Photographs of two species, which have been coded as TB75 and TB79, were excluded due to intellectual property (IP) sensitivity (photo courtesy: G. Turpin).

We apologize for this error.

